# Airplane tracking documents the fastest flight speeds recorded for bats

**DOI:** 10.1098/rsos.160398

**Published:** 2016-11-09

**Authors:** Gary F. McCracken, Kamran Safi, Thomas H. Kunz, Dina K. N. Dechmann, Sharon M. Swartz, Martin Wikelski

**Affiliations:** 1 Ecology and Evolutionary Biology, University of Tennessee, Knoxville, TN, USA; 2 Max Planck Institute for Ornithology, Radolfzell, Germany; 3 Biology, University of Konstanz, Konstanz, Germany; 4 Biology, Boston University, Boston, MA, USA; 5 Ecology and Evolutionary Biology, Brown University, Providence, RI, USA; 6 School of Engineering, Brown University, Providence, RI, USA

**Keywords:** bats, flight performance, airplane tracking, ground speed, wind modelling

## Abstract

The performance capabilities of flying animals reflect the interplay of biomechanical and physiological constraints and evolutionary innovation. Of the two extant groups of vertebrates that are capable of powered flight, birds are thought to fly more efficiently and faster than bats. However, fast-flying bat species that are adapted for flight in open airspace are similar in wing shape and appear to be similar in flight dynamics to fast-flying birds that exploit the same aerial niche. Here, we investigate flight behaviour in seven free-flying Brazilian free-tailed bats (*Tadarida brasiliensis*) and report that the maximum ground speeds achieved exceed speeds previously documented for any bat. Regional wind modelling indicates that bats adjusted flight speeds in response to winds by flying more slowly as wind support increased and flying faster when confronted with crosswinds, as demonstrated for insects, birds and other bats. Increased frequency of pauses in wing beats at faster speeds suggests that flap-gliding assists the bats' rapid flight. Our results suggest that flight performance in bats has been underappreciated and that functional differences in the flight abilities of birds and bats require re-evaluation.

## 1. Introduction

Flight evolved independently in birds and bats and the two lineages have met the demands of powered flight with numerous distinct adaptations [1–4]. It has been suggested that birds possess superior flight capabilities: specifically, that bird flight is more energetically efficient because aerodynamic force production results in more lift, and the wing morphology and wingbeat kinematics of bats result in greater induced drag and more complex wake structures [1–3,5,6]. External ears and facial structures associated with echolocation may also disrupt airflow over the bat body [2,7,8], although big ears may contribute positively with net aerodynamic force production [8]. Higher flight speeds and very long flight distances are reported more often from birds, supporting the generalization that bat flight is slower, less efficient and energetically more expensive [1,2,9,10]. However, models based on aerodynamic theory of fixed-wing aircraft [11,12], although incomplete for describing details of performance and for estimating the power required for flapping flight in birds and bats [13,14], predict relationships between wing shape and patterns of flight behaviour (such as preferred speed and manoeuvrability) that are supported by convergences between birds and bats, as observed in the field and laboratory. Most notably, birds and bats with longer and narrower wings (i.e. wings with high aspect ratios) typically fly faster and use more open habitats than those with shorter and broader wings. Because the scaling of wing span and wing area to body mass differs between the two groups, bats, which on average show lower wing loading (mass per area of wing surface), are expected to fly more slowly but to possess superior ability to manoeuvre compared with birds [1,3,11].

Common swifts (F. Apodidae, *Apus apus*) have high aspect ratio wings, high wing loading and streamlined body design. At a maximum ground speed of 31.1 m s^−1^, *A. apus* is the fastest flying bird on record to date [15]. Stooping raptors can reach even higher airspeeds of over 50 m s^−1^, but unlike horizontal powered flight, these flights employ gravity-assisted conversion of potential to kinetic energy. Similar to swifts, bats of the F. Molossidae have relatively high wing loading and high aspect ratio wings [11,16], they forage on insects in open airspace well above the ground, and are noted for their exceptionally fast flight. *Tadarida brasiliensis* (the Brazilian free-tailed bat) is among the best-known molossid species regarding its ability to employ rapid, long-distance flight. These small (approx. 12 g) bats emerge in large numbers from cave roosts in south-western North America, where they pursue insects to altitudes of over 1 km above ground level [17]. On clear nights, radar documents clouds of bats dispersing from roost locations at ground speeds of up to 14.7 m s^−1^ [18,19]. In the 1950s, flight speeds for *T. brasiliensis* were estimated from a helicopter to exceed 18 m s^−1^, and observations from the ground estimated maximum flight speeds of at least 26.8 m s^−1^ [20]. Although never replicated, these observations remain the fastest estimated horizontal flight for a bat.

To test the hypothesis that flight performance in the fastest flying bats may be underappreciated and comparable to that of birds, we documented the flight trajectories and ground speeds of seven free-flying *T. brasiliensis* during their nightly flights in open airspace above south-western Texas. To obtain flight speeds and information on wingbeat frequencies throughout their nightly flights, each bat carried a dorsally mounted, continuously emitting radio transmitter and was followed from an airplane for its complete nightly flight or until tracking was abandoned after 2.65 to greater than 5.5 h of continuous flight. We show that segments of rapid flight that include ground speeds faster than previously reported for any bat are interspersed with periods of slower flight and are part of continuous distributions of ground speeds. We further show that rapid flight is associated with increasing frequency of pauses in wingbeats implicating an association between flap-gliding and fast flight. Finally, we employ regional wind modelling to investigate effects of wind support on flight speeds and demonstrate that bats adjust their flight speeds in response to winds in a manner consistent with optimizing the costs of flight.

## 2. Material and methods

### 2.1. Airplane tracking

We tracked a total of seven bats, one bat per night, from the air between 8 July and 15 July 2009. For this, one person (M.W.) flew with a Cessna 172 from Garner Field Airport, Uvalde, Texas, towards the Frio Bat Cave while another person (T.H.K.) was stationed at the entrance of Frio Cave to await the nightly emergence of the bats. With the airplane in place, T.H.K. caught one bat with a hand net out of the column of emerging bats. We only tracked post-lactating females weighing 11–12 g. A 0.45 g radio transmitter was attached to the back of the bats using surgical glue (Skin Bond, Canada). Transmitters emitted a continuous signal to allow for the detection of wingbeat frequency as well as the position of the bat [21]. The glue was allowed to dry for a few minutes, and the bat was released back into the column of emerging bats. From then on, M.W. tracked the locations of the bats as follows.

Two RA-2AK-antennas (Telonics Inc., Mesa, USA) in the 160 MHz range were attached to the struts of the Cessna looking out horizontally, 90° from the flight direction of the plane. The cables running from the antennas to the cockpit were connected to an antenna switch, which in turn was connected to a LOTEK Biotrack wildlife receiver. The receiver was set to low signal amplification such that the signal from the transmitter would have maximum amplitude modification. M.W. then continuously switched back and forth between the left and right antenna while flying at an altitude of approximately 4200 feet above ground level (AGL). Whenever the signals from the left and right antenna were equal and of maximum amplitude, i.e. the plane was above the bat, a GPS point was taken. The effectiveness of this method has been proven repeatedly [22]. Its power relies upon both the human ear and the signal transmission/antenna reception pattern of radio transmitters. First, the human ear is a superb instrument to determine sound amplitude differences [23]. Second, as the aircraft passes directly over the transmitter, the gain of the antenna dipole is placed directly on the transmitter. The transmitter signal strength as perceived by the antennas shows an extremely steep peak at this point. Only at this point the radio signal completely saturates the receiver and the audio output of the receiver momentarily changes from a ‘beep’, or in our case, a continuous sinusoidal whistle, into a plodding or ‘thud’ sound or a deep, loud ‘humm’. For a continuous transmitter, the timing of the absolute peak signal level can be determined with a higher precision compared with a pulsed transmitter, because the specific time of maximum received power can be narrowed down to a few milliseconds by the human ear [23] compared with a sequence of one or two second-interspersed beeps in a pulsed transmitter. This more precise timing translates into an improved spatial accuracy. In our case, the signal-to-location matching was made easier as the pilot also conducted the radio telemetry, which is not usually the case in aerial telemetry. As the trajectory of the bat was unknown, the pilot tried to keep up with the bat in speed and direction to precisely locate the bat as often as possible, which occurred about every 2–5 min (table 1).

**Table 1.** Metrics for flight trajectories of seven Brazilian free-tailed bats.

	no. locations	total travel distance (km)	total duration (h)	mean ± s.d. distance between consecutive locations (m)	max distance between consecutive locations (m)	min distance between consecutive locations (m)	mean ± s.d. time lag between consecutive locations (s)	mean ± s.d. ground speed (m s^−1^)	median ground speed (m s^−1^)	max ground speed (m s^−1^)
bat 1	116	100.5	4.03	873.72 ± 461.67	1980.5	122.6	126.26 ± 65.76	9.82 ± 8.58	6.98	39.65
bat 2	64	53.8	3.33	854.57 ± 454.94	2266.1	37.5	190.47 ± 109.50	6.77 ± 6.49	3.69	27.23
bat 3	54	62	2.65	1170.72 ± 692.29	3593.4	231.1	180.07 ± 132.22	10.3 ± 7.95	8.28	30.71
bat 4	86	71.4	4.73	840.66 ± 739.49	5189.3	119.9	200.14 ± 225.48	5.35 ± 5.08	4.41	38.73
bat 5	117	102.4	5.54	883.03 ± 603.52	4039.5	42	172.06 ± 84.50	6.45 ± 5.88	4.7	29.71
bat 6	56	88	3.75	1600.84 ± 972.11	5688.5	174.5	245.54 ± 191.36	10.28 ± 10.02	6.63	38.41
bat 7	71	159.8	4.58	2316.06 ± 2243.63	12799	0	239.13 ± 309.27	17 ± 12.08	14.33	44.5

Each bat was then followed as long as it remained actively flying throughout the night and tracking was only stopped when the bat returned to the cave 3–5 h later or, in one case, when one bat entered an abandoned building in Uvalde, TX (located from the air to the nearest 50 m using the method described above, allowing us to get a visual confirmation of the bat inside the building) or, in another, when a bat that had been on a very long southward foraging flight started to return towards the colony. Bats could only be tracked for a maximum of 6.5 h due to the fuel needs of the Cessna, always leaving 2 h reserve flight capacity. The bats that had returned to the colony were located within the roost during subsequent days and bats retained the transmitter for 2–5 days, but then presumably groomed it off.

### 2.2. Locational accuracy of tracking methodology

Using the same tracking equipment, the potential error in determining the true location was measured in Germany in March 2015. A radio transmitter was attached to the roof of a car carrying a GPS locator and its position was tracked from a Cessna plane at approximately 4000 feet AGL. The car driver and the plane pilot (M.W.) did not know each other's whereabouts, thus simulating the bat tracking. The car could not be identified from the air at this altitude and was driven at speeds from 20 to 180 km h^−1^, simulating *T. brasiliensis* foraging movements and very fast directional distance movements. The radio transmitter on the car was localized as described above for the bats and a total of 12 locations were taken during a 1 h flight. To estimate the effect of the measured locational error on speed estimates, we randomly repositioned each location 1000 times by a distance drawn from a random normal distribution with the measured mean and standard deviation of errors and recalculated speed.

### 2.3. Wingbeat frequency

The radio signal of the bat transmitter was recorded on a laptop for 30 min to 1 h, starting approximately 15 min after the bat was released, such that the wingbeat frequency and amplitude could be analysed later, based on the changes in radio signal. For a continuous transmitter, the slight pressure changes induced onto the body of the transmitter by the beating of the wings in a bat or bird (i.e. the up-and-down movements of the animal's body) result in a change in transmitted signal amplitude, which can be easily discerned by the human ear or analysed via audio programs. A graphical description is shown in Cochran *et al.* [21]. Continuous wingbeat frequency, in which bats flapped continuously without missing a beat, was calculated. When the amplitude of the recorded radio signal did not change, i.e. when there was no wingbeat, the numbers of skipped beats were interpolated, as was previously done for songbirds [21]. Numbers of skipped beats were then counted for randomly selected 5 s segments from time periods when ground speeds were at selected values, and wingbeat pause frequencies [21] were examined in relation to ground speeds. The data were transformed to obtain normal Gaussian error distribution as follows: log(wingbeat pauses + 1) and log(ground speed).

### 2.4. Flight statistics

We investigated the flight trajectories, and used the statistical and programming environment R (v. 3.0.2) and the package ‘move’ to calculate, duration, distances and, from those measures, speed for each track segment and total and net displacement for each bat's nightly flight. Distances were based on great circle distances from the coordinates of the trajectories taking into account the curvature of the Earth surface.

### 2.5. Wind annotations

We annotated the wind direction and speed for the positions of the bats using a weighted distance interpolation [24] in the spatial and a linear interpolation in the temporal domain using the ‘EnvDat’ tool [25] on movebank.org (data from EnvDat on movebank.org). The data source was the National Centers for Environmental Prediction (NCEP) providing the North American Regional Reanalysis (NARR), a high-resolution wind model with an approximate 0.3° (32 km) grid resolution at the lowest latitude, available through the public weather-reanalysis data portal of the National Oceanic and Atmospheric Administration (NOAA). Although large-scale weather models cannot capture the dynamics and turbulence structure of any single place and time, evidence suggests that we can expect large-scale models to correlate with local conditions [24]. Because the flight altitude of the bats while on the wing was unknown, we repeated the analysis of the effect of wind on speed from 10 m AGL to approximately the flight altitude of the airplane. Thus, we calculated surface level winds at 10 and 30 m AGL, corresponding with the depth of the first atmospheric layer in the weather models, as well as winds at the pressure levels from 1000 to 850 mbar at steps of 50 mbar (electronic supplementary material, table S1), which corresponds roughly to winds modelled from sea level to about 1500 m above sea level (ASL).

### 2.6. Effects of wind support and cross wind

To model the effect of wind direction and speed on the flight of the bats, we restricted the analysis to those segments where ground speed (speed relative to ground) was measured to be faster than 2 m s^−1^. This ensured we excluded the initial and final phases during which the bats were stationary, presumably roosting, and therefore unaffected by the climatic conditions. From wind speed and direction, we derived wind support, which is the length of the wind vector at any known location of the bat in the bats' travel direction towards the next location. Positive wind support values represent tail wind and negative values head wind. Cross wind represented the length of the wind vector perpendicular to the bats' direction of travel irrespective of the side and was expressed as a positive value. Using wind support and cross wind, we calculated air speed (speed relative to air; electronic supplementary material, figure S1) and modelled the influence of wind support and cross wind on ground speed as well as wind support and cross wind on air speed using a generalized additive mixed model. The statistical models accounted for individual differences by using a mixed model design and accommodated the complex correlation structures of time, with linear fixed effects of cross wind and wind support. A log transformation of air and ground speed was necessary to achieve normal error distribution of the residuals. To account for the temporal (and thus also spatial) autocorrelation in the residuals, we included a smooth term with the time since beginning of the flight in all models. The complexity of the smooth term, i.e. the number of effective degrees of freedoms used, was initially set to 50 and made subject to an extra penalty, so that the complexity of the smooth term was part of the model fitting procedure where it could be penalized to zero, selecting for the most parsimonious model term. The effective degrees of freedom used for fitting the smooth term (time) was, in most models (of the different height and pressure levels), reduced to less than 3. Modelled wind and air speed relationships were qualitatively similar at all heights (electronic supplementary material, table S1), and are described here for 30 m AGL.

To further investigate the potential effects of winds, we downloaded wind data (reported every 20 min) from the closest meteorological station, Garner Airfield, TX, approximately 40 km from the roost (http://gis.ncdc.noaa.gov/map/viewer/; http://www.srh.noaa.gov/data/obhistory/KARV.html). We binned the weather station data on wind speed and direction and our ground speed measurements for bats into hourly bins and compared the maximum values of ground speed and maximum wind speed in the same hour.

## 3. Results

**Figure 1.**
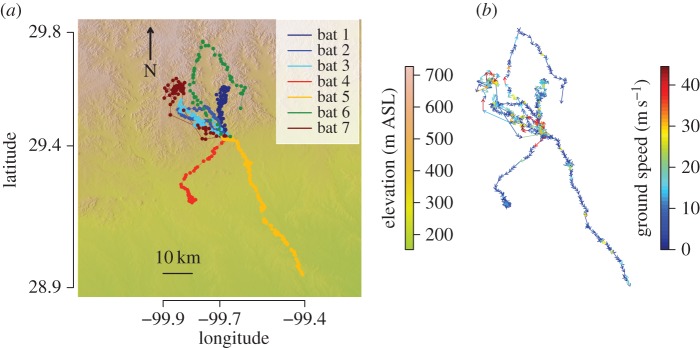
Flight trajectories and ground speeds. (*a*) Consecutive radio-tracking fix locations are indicated by circles. Detailed metrics were calculated for the trajectories based on the distances and times between fixes (table 1). Colours and individual identifications are consistent across all figures. Background colours and contour lines indicate topography in metres ASL. (*b*) Colour-coded ground speeds with arrows plotted on the trajectories to illustrate direction and speed measured for each segment. High speeds were distributed throughout the bats' flight trajectories.

We tracked single bats for cumulative distances of 54 to 160 km per night (figure 1 and table 1). Mean distances between consecutive radio-fix locations (flight track segments) ranged from 841 to 2316 m, with mean time lags between fixes ranging from 2.1 to 4.0 min (table 1). There was no evidence for night roosting or interruptions in the flights, as speeds were consistently high over the durations of all flights. Low speeds were only recorded at the beginning and end of each trip.

**Figure 2.**
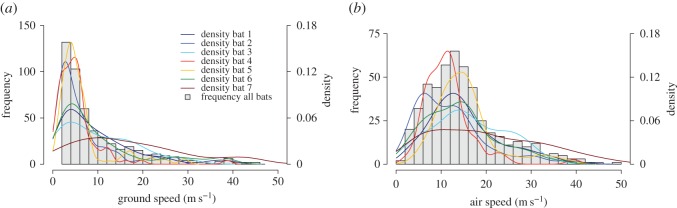
Flight speed distributions for bats. (*a*) Frequency distribution for the measured ground speeds of all bats and their kernel densities for each individual in metres per second. (*b*) Frequency distribution of air speeds for all bats and their kernel densities for each individual in metres per second.

The distribution of ground speeds was highly skewed (figure 2*a*), with medians for individual bats ranging from 3.7 to 14.3 m s^−1^, and an overall median ground speed for the seven bats of 5.7 m s^−1^. However, all bats achieved ground speeds above 25 m s^−1^, and five of the seven bats achieved ground speeds above 30 m s^−1^ (table 1). After culling one apparent outlier of 60.1 m s^−1^ that was more than 15 m s^−1^ above the next fastest speed of 44.5 m s^−1^, we confirmed that speeds above 25 m s^−1^ belong to the same continuous distribution as all ground speeds (electronic supplementary material, figure S2), demonstrating the fast speeds are not artefacts of measurement bias related to the distances or time lags between flight track segments. Individual maximum ground speeds were achieved during brief periods within single track segments and ranged from 27.2 to 44.5 m s^−1^ (figure 1 and table 1).

Airplane tracking of the GPS-equipped, radio-tagged car moving at the same speeds as measured for the bats revealed a locational accuracy over all speeds of 89.9 ± 50.0 m (mean ± s.d.). Bootstrap analysis of the potential effects of this error revealed that the 95% confidence limits for estimated speeds increased with increasing measured speed (95% CI at the maximum speed of 45 m s^−1^ was 38.6 and 54.3 m s^−1^; electronic supplementary material, figure S3).

**Figure 3.**
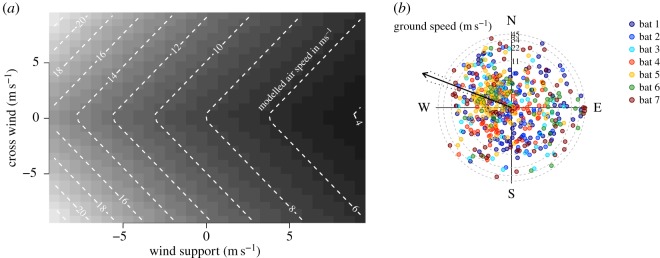
Response of bats to winds. (*a*) Modelled air speed (metres per second) isoclines in reference to wind support and cross winds based on wind data at 30 m AGL. Positive wind support values are tail wind and negative values are head wind. Cross winds are the length of the wind vector perpendicular to the bats' direction of travel. (see electronic supplementary material, table S1, for additional information). (*b*) Ground speeds and the azimuth of flight directions for seven bats. Prevailing winds on all nights were E to SE (90° to 135°). Faster ground speeds were not associated with prevailing winds.

Modelling the effects of winds on the measured ground speeds of bats shows that bats responded to winds by lowering airspeed as wind support increased, and increasing airspeed as cross winds increased (figure 3*a*). Our wind models indicate that all seven bats exceeded air speeds of 25 m s^−1^ (figure 2*b*), and reveal no pattern of bats travelling at higher ground speeds in the direction of the prevailing winds, or any particular direction (figure 3*b*).

The analysis of wind data from the closest meteorological station showed that ground speeds above 40 m s^−1^ were measured during hours where the maximum reported wind speeds were less than 6 m s^−1^, including during hours with no reported wind (electronic supplementary material, figure S4). A linear model showed no statistically significant relationship between maximum ground and maximum wind measured during the same hour (slope = −0.7 ± 0.72, *F*_1,34_ = −0.94, *p* = 0.34). If anything, the relationship tended to be negative, in line with our finding from the North American regional atmospheric models.

**Figure 4.**
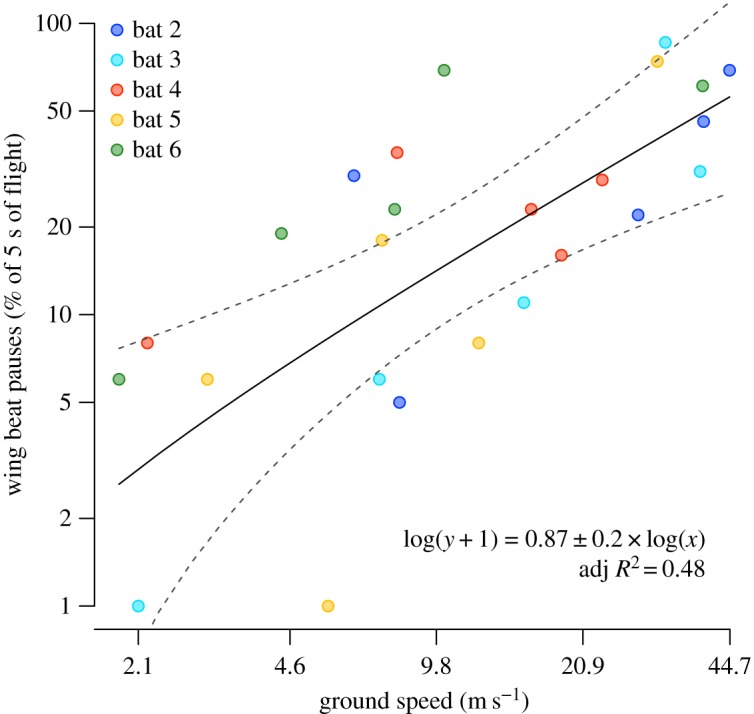
Ground speed versus wingbeat pauses. Wingbeat pauses increase with increasing ground speeds (both measurements shown on logarithmic scales), suggesting that bats are more likely to skip wingbeats and glide at very fast speeds. The linear relationship is based on a sample size of 25 observations (five sections of the flight of five individuals). The data were transformed to obtain normal Gaussian error distribution as follows: log(wingbeat pauses + 1) and log(ground speed). The model (*F*_1,23_ = 23.1, *p* < 0.0001, adj *R*^2^ = 0.5) showed a highly significant positive linear relationship between wingbeat pauses and ground speed as indicated in the figure (log(ground speed) effect size ± s.e. = 0.87 ± 0.18, *t* = 4.8, *p* < 0.0001). The model intercept was non-significant.

We obtained wingbeat frequency measurements from five of the seven bats flying at the range of ground speeds that we document. Our data demonstrate a strong positive association of higher ground speeds with increasing wingbeat pauses (figure 4).

## 4. Discussion

Generalizations concerning their comparative flight abilities acknowledge that the full range of flight performance in bats is presently understudied relative to that in birds [1,3,6]. Molossid bats, including *T. brasiliensis,* occupy an extreme morphospace among bats [3,11,16] and *T. brasiliensis* exploits an aerial niche similar to that of swifts and swallows, leading us to expect efficient, high-speed flight performance in these bats. This expectation is supported by particle image velocimetry (PIV) investigations of wind tunnel flight in bats. To date, PIV has been employed to investigate flight in seven of the over 1300 currently described species of bats [26]. Four of these, *Glossophaga soricina, Leptonycteris yerbabuenae* [5,27], *Cynopterus brachyotis* [28] and *Carollia perspicillata* [14], are nectar/fruit-eating bats with broad low aspect ratio wings that fly in cluttered habitats and are capable of hovering, and two are small broad-winged insectivores [8,29]. The seventh species is *T. brasiliensis*. In general, the six broad-winged species produced the complex wake structures typically associated with lower lift-to-drag ratios and reduced span efficiency in bats [5,8,27,28,29]. By contrast, *T. brasiliensis* demonstrated wake architecture and three-dimensional wing kinematics much more similar to swifts [6]. As wind tunnel airspeeds increased, several kinematic parameters changed to simultaneously reduce drag and inertial forces, improving efficiency. Consequently, the flight dynamics of birds and some bats may not be as distinct as supposed from previous comparisons based on wake structure and kinematics of the broad-winged, primarily nectar/fruit-eating bats [6]. Moreover, top speeds of this study, of no more than 9 m s^−1^, are unlikely to have challenged *T. brasiliensis*' true flight capabilities.

While existing studies of wake architecture and kinematics indicate that the full range of bat flight has yet to be described, other studies suggest that the efficiency of bat flight also may be greater and more similar to birds than commonly assumed. Direct measures of the metabolic costs of flight in similarly sized animals provide evidence that metabolic power of hovering is significantly less and, hence, aerodynamic efficiency greater, for bats than for birds or insects [30,31]. In addition, data compiled for bat and bird species of comparable body mass suggest no significant differences in mass-specific metabolic power in relation to velocity for forward flight up to 10 m s^−1^ [32].

The adjustments of air speed in response to wind support that we document (figure 2*b* and figure 3*a*) are similar to those previously observed for birds, insects and other bats [33], and are consistent with models for optimal orientation to winds [34]. As a result of these adjustments, our analyses indicate that bats maintain similar ground speeds regardless of the strength of wind support (figure 2*a,b*) [33]. The response of bats to winds (figure 3*a*), and the lack of an association between ground speeds and the directions of prevailing winds (figure 3*b*) suggest that tailwinds do not account for the fast ground speeds that we document. However, within the spatial and temporal resolution of our wind analyses, we cannot rule out the possibility that fast ground speeds were assisted by fluctuating local wind gusts, perhaps enhanced by the hilly terrain (figure 1). In the absence of detailed and time-resolved knowledge of flight altitudes and local wind conditions, our understanding of the behavioural context and mechanistic basis of these high-speed flights is currently limited.

Flap-gliding, where flying animals alternate between powered flapping wingbeats and gliding, is documented for many birds [21,35], but for only a few bats [36,37]. In a recent aerodynamic model, lift-to-drag ratio increased during gliding in common swifts, reducing the energetic costs of continuous flapping flight [35]. Recent work also indicates that energetic benefits may accrue from flap-gliding by reducing costs of muscle activation during downstroke and reducing the need to compensate for loss of weight-support during upstroke [38]. Flap-gliding in birds is often associated with steady, direct flight [38]. One previous study showed captive bats to increase flight speeds during steep downward glides [36], but there are, to date, no data on the energetics and flight performance of flap-gliding in bats. Our data associating higher ground speeds with wingbeat pauses (figure 4) [21] suggest that flap-gliding assists the rapid flight of Brazilian free-tailed bats, but data are as yet insufficient to test hypotheses relating flap-gliding to flight speeds.

Our tracking of the complete or nearly complete flights of seven *T. brasiliensis* results in a unique dataset. Whereas flight speeds of free-flying birds and bats are typically reported as single parameter values or as averages with associated standard deviations [9,11,39], we report continuous distributions of flight speeds over periods of several hours. Recordings from tracking radar and high-speed video allow collection of multiple flight tracks and a distribution of flight speeds, but these tracks are typically short samples (a few metres) over brief time periods (fractions of seconds to several minutes) of individual flight trajectories [15,37] (but see Henningson *et al*. [40]). The flight speed record for *A. apus*, for example, was obtained from high-speed stereo camera recordings of 25 flight sequences (average distance covered = 1.95 m) at low altitude. The maximum potential wind speed was estimated at 2.6 m s^−1^, with horizontal flight speeds ranging from 11.9 m s^−1^ to 31.1 m s^−1^ [15].

The average flights speeds of 5.4 to 17.0 m s^−1^ for our seven bats are within the range of reports for several bird and bat species [37,39,41], including *T. brasiliensis*. In fact, the median flight speed of our fastest *T. brasiliensis* (14.3 m s^−1^; table 1) is nearly identical to the maximum median flight speed reported for the European free-tailed bat, *Tadarida teniotis* (13.9 m s^−1^) [41], another molossid species similar to *T. brasiliensis* in morphology and flight ecology. The density of our tracking data demonstrate that in *T. brasiliensis* these average speeds are punctuated with spurts of rapid flight (figure 1*b*) that are faster than previously recorded for any bat. Although different measurement techniques and unaccounted effects of local winds may preclude direct comparison, these speeds also exceed those currently reported for the fastest flying birds [15]. Repeated bouts of high-speed locomotion represent a strategy that has evolved frequently, and bouts of such high speed may not be unusual. For example, cheetahs achieve top running speeds of 25.9 m s^−1^, but their fastest runs account for only a fraction of the average total distance travelled [42]. Because ground speeds for bats were measured from the horizontal component of distances travelled during 2.1–4 min intervals, the maximum speeds that we report here are undoubtedly conservative estimates of the speeds of which these bats are capable. Our results suggest that further investigation of the full range of flight performance in bats will be rewarding.

## Supplementary Material

Electronic supplementary material includes: Figure S1 Figure S2 Figure S3 Figure S4 Table S1

## Data accessibility

The data used in this study are publicly available on Movebank (movebank.org, study name Flight speed in Brazilian free-tailed bats (data from McCracken *et al.* 2016) and are published in the Movebank Data Repository (doi:10.5441/001/1.td71sn54).
