# Supplementary material for: Airplane tracking documents the fastest flight speeds recorded for bats
Source: R Soc Open Sci. 2016 Nov 9;3(11):160398. doi: 10.1098/rsos.160398 (PMC5180116; doi:10.1098/rsos.160398)
Supplement: Electronic supplementary material includes: Figure S1 Figure S2 Figure S3 Figure S4 Table S1 [file rsos160398supp1.docx]

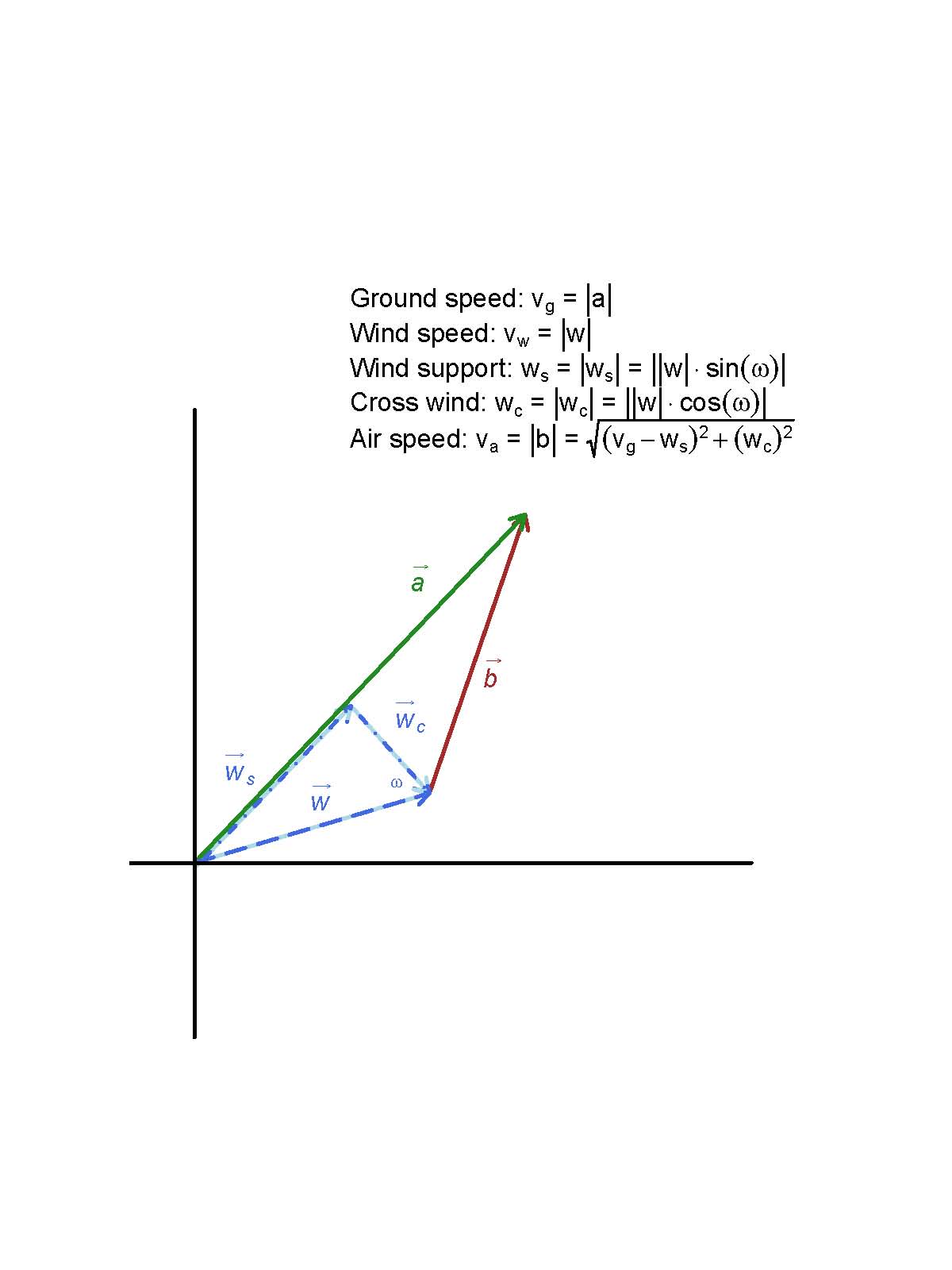


**Figure S1.** Schematic representation of the calculated measures, where ‘a’ represents the vector of a bat's movement relative to the ground. Its length is v_g_ . Wind support (w_s_) is the length of the wind vector in the direction of a and cross wind (w_c_) the length of the perpendicular component. Finally, airspeed (v_a_) is the speed of the bat relative to the wind and can be calculated as given


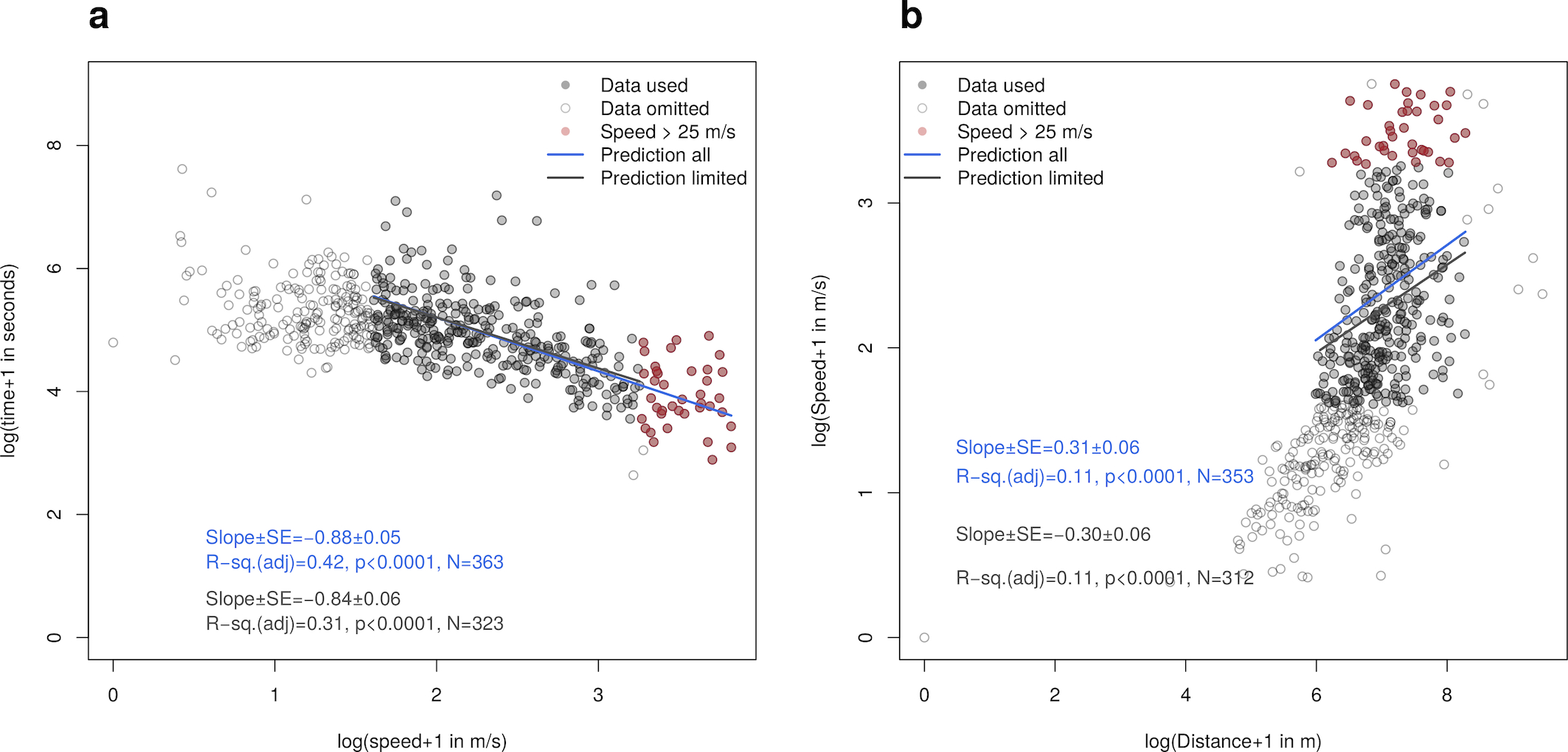


**Figure S2**. **a,** Distribution of the log of all measured ground speeds versus log distance between consecutive location fixes, and distribution of the log of ground speeds above 25 m s^-1^ versus log distance between consecutive location fixes. **b,** Distribution**s** of the log of all measured ground speeds and ground speeds above 25 m s^-1^ versus the log of the time-lags between consecutive location fixes. Identical slopes were observed for both sets of speeds, demonstrating that the faster speeds belong to the same distributions as all speeds.


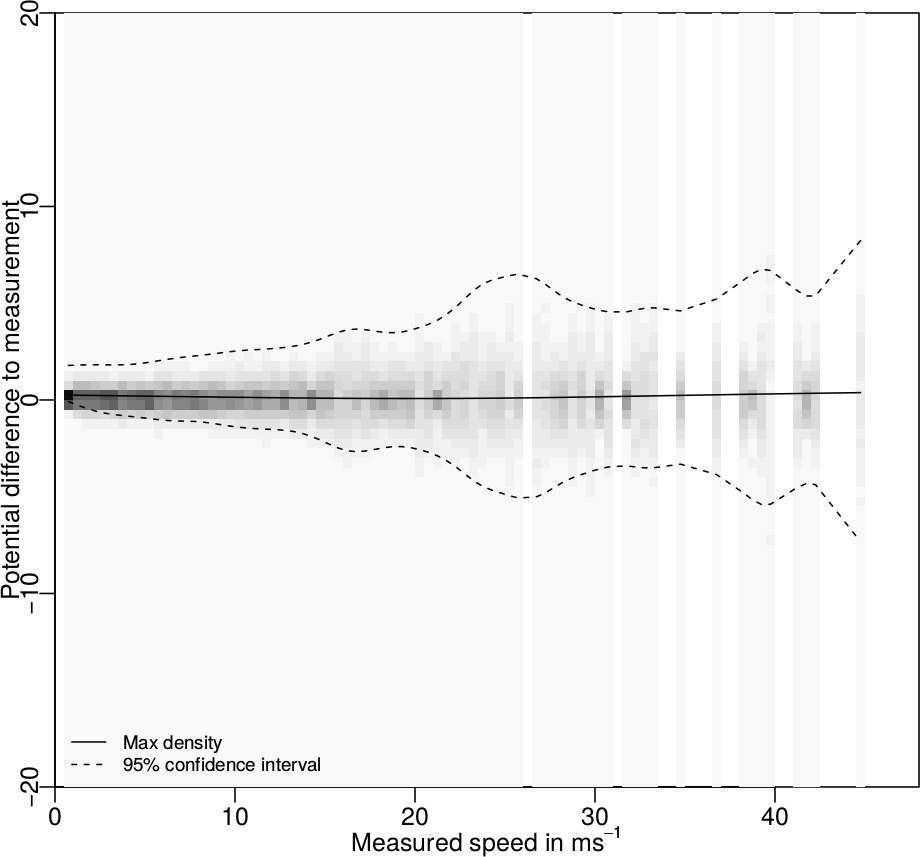


**Figure S3**. Simulated change in ground speed as a consequence of the error in determining the true position based on the measured mean error of 89.9 meters and the standard deviation of ± 50.0 m. Using 1000 random repositions of the measured locations by drawing from a normal random distribution, we estimated the confidence interval for each speed measurement. The simulation reveals that the width of the confidence interval increased with increasing measured speed, suggesting that segments of the trajectory with high measured speeds were more susceptible to error. However, the magnitude of the estimated change was comparably low given the estimated speeds. The white lines mark the upper and lower boundaries containing 95% of the changes when locations were repositioned randomly according to the measured error and the dashed line represents the peak probability.


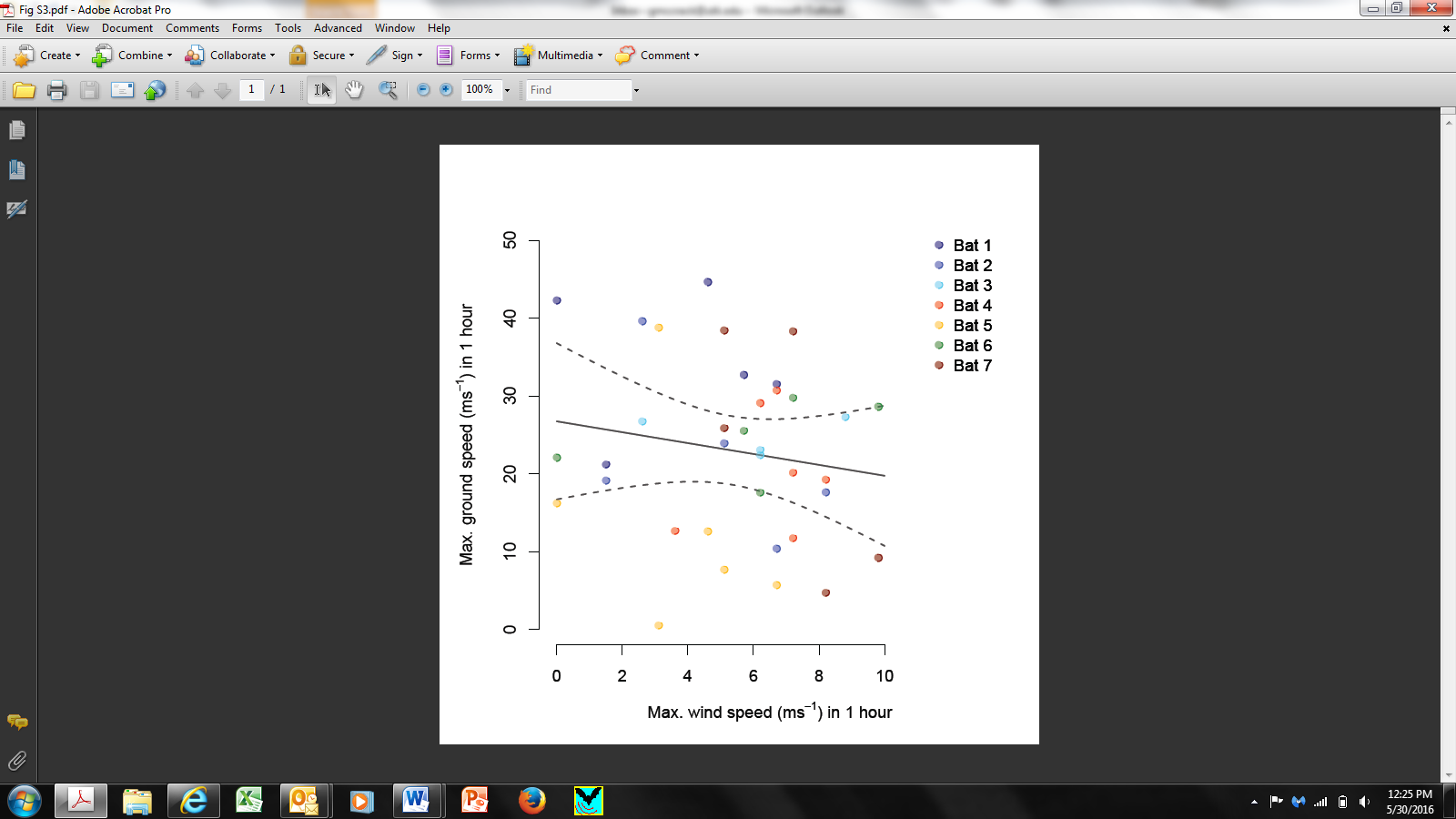


**Figure S4.**  Maximum ground speed measured in a specific hour versus the maximum wind speed measured at the closest weather station during the same hour indicated no relationship between wind speed and ground speeds achieved. The linear relationship (solid line represents the prediction and the dotted lines are the standard error) was not significant.

**Table S1**: Estimated effects of cross wind and wind support on log (air speed + 1) in bats based on generalized additive mixed models (GAMM) for different heights and pressure levels. To adjust for individual differences in air speed, individual ID was chosen as a random effect. The distribution of the error was Gaussian and a smooth term with time lag between observations was included as a covariate to account for temporal autocorrelation of the observations. The effective degrees of freedom used for the smooth term were subject to an extra penalty, meaning that the smoothing parameter estimation, or the complexity of the relationship between the time lag and the predictive power of the model, were part of the model fitting process. Therefore, the smooth term was reduced to the minimum amount of necessary complexity as indicated by the change in model fit. All parameter estimates (intercept, cross wind and wind support) were significantly different from zero with significance levels well below 0.0001. The adjusted R^2^ indicates roughly the model fit and is influenced by the degrees of freedom used by the fixed effects and the varying number of degrees of freedom of the optimized smooth term. Sample size was for all models 496 segments, with the same seven individuals as random effects.

|  | Estimates±s.e.m. | | | Smooth term | | | |  |
| --- | --- | --- | --- | --- | --- | --- | --- | --- |
| Pressure level | | Cross wind | Wind support | Intercept | edf | F | p | adj R^2^ |
| 10 m above ground | 0.04±0.01 | -0.08±0.006 | 2.19±0.09 | 2.9 | 9.8 | <0.0001 | 0.23 |  |
| 30 m above ground | 0.04±0.01 | -0.06±0.004 | 2.25±0.08 | 2.7 | 7.7 | 0.001 | 0.31 |  |
| 1000 mbar (110 m asl) | 0.04±0.008 | -0.06±0.004 | 2.28±0.08 | 2.3 | 4.3 | 0.01 | 0.33 |  |
| 950 mbar (540 m asl) | 0.04±0.008 | -0.05±0.003 | 2.31±0.07 | 2.2 | 3.0 | 0.04 | 0.34 |  |
| 900 mbar (980 m asl) | 0.03±0.006 | -0.05±0.003 | 2.36±0.06 | 1.9 | 1.7 | 0.2 | 0.4 |  |
| 850 mbar (1450 m asl) | | 0.04±0.007 | -0.05±0.003 | 2.32±0.07 | 2.1 | 3.5 | 0.03 | 0.36 |

Model: log (air speed + 1) = Cross wind + Wind support + s (time lag)
